# Supplementary material for: Connective differences between patients with depression with and without ASD: A case-control study
Source: PLoS One. 2023 Aug 15;18(8):e0289735. doi: 10.1371/journal.pone.0289735 (PMC10427005; doi:10.1371/journal.pone.0289735)

Supplementary Information

We uploaded the signal-to-noise ratio (SNR) of each preprocessing section and obtained the SNR at each stage of preprocessing (preprocessing, post-realignment, post-normalization, and post-smoothing) using the compute-SNR-brain program (Jan Valošek, Pavel Hok; fMRI laboratory Olomouc; https://github.com/valosekj/compute_snr_brain).

The analysis method is described below.

Script performs the following:

1. Reorientation of the input image to match the approximate orientation of the standard template image (MNI152)
2. Brain extraction
3. Tissue-type segmentation (white matter, gray matter, CSF)
4. Creation of white matter mask
5. Creation of four cubic ROI places in the superior corners of the input 3-D image representing noise
6. Computation of the SNR using the following equation:

   SNR = mean_wm (white matter) / mean_noise

Each SNR was analyzed using R (4.2.3).

The SNR increased with each stage of preprocessing. We analyzed the univariate type III repeated-measures analysis of variance assuming sphericity among each SNR. We identified significant differences among them (F value 131.34, $p<0.001$). Pairwise comparisons using the paired t-test adjusted by Bonferroni suggested significant differences in all SNRs, except for the difference between the SNRs of preprocessing and post-realignment.

This method for determining the SNR is based on the methodology described previously:

Nagaki A, Onoguchi M, Matsutomo N. Clinical validation of high-resolution image reconstruction algorithms in brain 18F-FDG-PET: effect of incorporating Gaussian filter, point spread function, and time-of-flight. Nucl Med Commun. 2014;35:1224-1232. doi: 10.1097/MNM.0000000000000187.

Supplementary Table 1: Signal-to-noise ratio (SNR) of each processing process

		Pairwise comparisons using paired T-test		
	SNR Mean [SD]	Preprocessing p-value	Post-realignment p-value	Post-normalization p-value
Preprocessing	1.62 [0.46]	-	-	-
Post-realign	1.65 [0.48]	1	-	-
Post-normalization	25.90 [10.37]	5.60E-09	5.80E-09	-
Post-smoothing	31.10 [11.12]	6.20E-10	6.20E-10	2.10E-06

Supplementary Figure

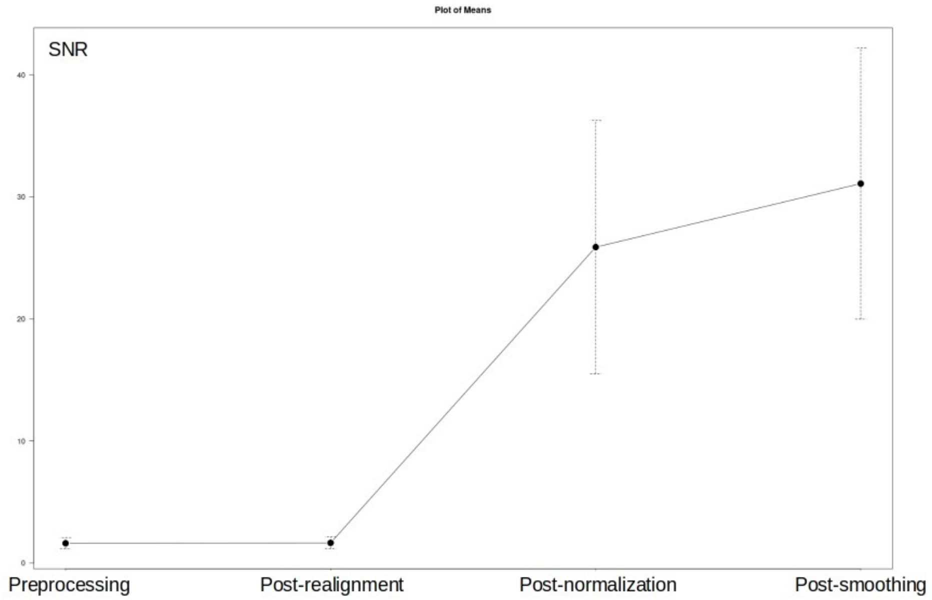

Supplement: S1 File — (PDF) [file pone.0289735.s001.pdf]
