# Supplementary material for: Connective differences between patients with depression with and without ASD: A case-control study
Source: PLoS One. 2023 Aug 15;18(8):e0289735. doi: 10.1371/journal.pone.0289735 (PMC10427005; doi:10.1371/journal.pone.0289735)
Supplement: Attachment — Submitted filename: Connective Diff-Comments.docx [file pone.0289735.s002.docx]

**Title: Connective differences between autism spectrum disorder with depressive state and depression: case-control study**

1. How the authors describe the resting state of ASD individuals? Since the people with ASD has different comorbidities and exhibit restlessness in some individuals,
2. Authors pls check for the abbreviations for eg:-DSM 5 is repeated .It can be defined at initial stage itself.
3. Methodology can be given in detail using a block diagram or pictorial representation
4. Technical information is lagging in the manuscript. Such as pre-processed images its metrics and equation related to filtering
5. There is no proof for pre-processing of the functional and structural images and the metrics can be specified.
6. Did the ASD participants were assisted by their caregivers?
7. ASD individuals don’t adjust to social setting. Pls mention the challenges in recording rs fMRI
8. Figure 1 and 2 can be cited with more information.
