# Supplementary material for: Connective differences between patients with depression with and without ASD: A case-control study
Source: PLoS One. 2023 Aug 15;18(8):e0289735. doi: 10.1371/journal.pone.0289735 (PMC10427005; doi:10.1371/journal.pone.0289735)
Supplement: Attachment — Submitted filename: revise_comment_to_reviewer_revise.docx [file pone.0289735.s003.docx]

5. Review Comments to the Author

Please use the space provided to explain your answers to the questions above. You may also include additional comments for the author, including concerns about dual publication, research ethics, or publication ethics. (Please upload your review as an attachment if it exceeds 20,000 characters)

Reviewer #1: a) the manuscript can provide further more information regarding the Autism Spectrum Disorder with depressive state and with depression for clarity of the topic and need to do the research. the reviewer wasn't able to capture the clear information regarding the ASD with depressive state and depression. it is understood from science that brain structural differences are seen in persons with ASD and without ASD and this needs to correlated as the study is done with the help of MRI.

**Response:** We thank the reviewer for this comment. In response, we have performed voxel-based morphometry and added the following sentences to the Methods, Results, and Discussion sections.

Methods

***“Structural analysis***

Voxel-based Morphometry

We used SPM12 to estimate differences between depression and ASD with depressive state. Preprocessed structural images were smoothed with a 6-mm Gaussian kernel of FWHM to increase the S/N ratio.

Post-processed structural images were divided into two groups: ASD with depressive state and depression. First, we constructed a design matrix to adopt the general linear model (GLM) and identify regions significantly related to the differences between the two groups. We also performed estimation to validate the independently distributed residuals. Finally, we analyzed the two-sample t-test using the standard parametric procedure to test the hypothesis.[21]”

Results

“***Volumetric findings***

There was no significant difference between ASD with depressive state and depression.”

Discussion

“We performed voxel-based morphometry using SPM12 to compare the structural differences between ASD and depression.[21] Our results showed no significant structural differences between these two groups, although previous studies demonstrated variable structural abnormality in ASD subjects presenting with increased and decreased volumes in specific areas of the brain.[36-39] ASD subjects in previous studies varied in both age and clinical background (comorbidity, educational and genetic profiles). Compared with healthy controls adult ASD subjects, Ecker et al showed that adult ASD subjects had increased gray matter volume in the anterior temporal and dorsolateral prefrontal regions and decreased volume in the occipital and medial parietal regions, despite there being no significant difference in whole brain volume.[36] In the present study, ASD subjects showed no structural differences compared to subjects with depression. However, subjects with depression were known to display significant volume reductions compared to healthy controls, especially in the hippocampus and amygdala.[40-41] We assumed that the differences in morphological alterations in each study could be due to differences in clinical background. Therefore, our results indicated some structural abnormality in ASD subjects that should be reexamined in healthy controls.”

b) as far as ASD condition is concerned, the males are more affected by ASD than females. the article should have pronounced sex differences, since females experience twice as much depression as males.

**Response:** We thank the reviewer for this comment. We added following paragraphs in the Discussion section.

“In this study, the numbers of male and female subjects were equal in both the groups. Although there were slightly more male subjects with ASD and slightly fewer male subjects with depression, there was no statistically significant difference in gender distribution. In general, ASD is more than 4 times more common among males than among females, and depression is twice as common among females.[22-24] The equal number of males and females in this study was considered to match that in a previous study because of the opposite gender distribution between ASD and depression. However, the sample size in this study was too small to analyze gender differences. In the case that no gender differences were found in the population presenting with depression, other psychiatric disorders may need to be considered.”

c) As a Rehabilitation Professional-Educationists, Psychologists look for the behavioural output of the subjects. it is not revealed in the study and the reviewer has limitations in further reviewing the manuscript.

**Response:** We thank the reviewer for this comment. At our institution, patients referred from other institutions are examined, psychologically tested, and diagnosed clinically by an experienced psychiatrist. In this study, patients showed symptoms of depressive state, as shown in the inclusion criteria. However, subsequent physical examination and psychological testing revealed that the depressive state was associated with ASD rather than depression. Many patients received rehabilitation and treatment at the referral institution after the diagnosis was made. It was therefore difficult to consider outputs in more detail. We would appreciate the reviewer’s understanding on this matter.

Reviewer #2: Title: Connective differences between autism spectrum disorder with depressive state and depression: case-control study

1. How the authors describe the resting state of ASD individuals? Since the people with ASD has different comorbidities and exhibit restlessness in some individuals,

6. Did the ASD participants were assisted by their caregivers?

7. ASD individuals don’t adjust to social setting. Pls mention the challenges in recording rs fMRI

**Response:** We thank the reviewer for this comment. As pointed out, MRI examinations are considered difficult for patients with ASD, ADHD, and PDD, and ASD was reviewed. All participants in this valuable review were under 20 years of age, and most were children around 10 years of age. In contrast, our target patients were aged over 20 years and were willing to participate in this MRI study. In addition, he presented to the hospital in a depressed state, which is also considered to be the reason why there was no interruption due to movement during the examination and no deterioration of the image. All patients did not require caregiver attendance. However, as the reviewer pointed out, testing is expected to be difficult in general; we would therefore like to state in the Discussion section that the subject in this case was biased toward ASD who showed depressive state. We have revised the relevant paragraphs in Methods and Discussion sections according to these comments.

2. Authors pls check for the abbreviations for eg:-DSM 5 is repeated .It can be defined at initial stage itself.

**Response:** We thank the reviewer for this comment. We have modified the manuscript accordingly.

3. Methodology can be given in detail using a block diagram or pictorial representation
4. Technical information is lagging in the manuscript. Such as pre-processed images its metrics and equation related to filtering
5. There is no proof for pre-processing of the functional and structural images and the metrics can be specified.

**Response:** We thank the reviewer pointing this out. We added details on preprocessing accordingly. In addition, we created a block diagram explaining the processing method conducted in our study. Regarding the metrics, we did not add a corresponding diagram and explanation because they are described on the relevant web page.

8. Figure 1 and 2 can be cited with more information.

**Response:** We thank the reviewer for this comment. We have inserted the necessary citations in the figures accordingly.
