# Supplementary material for: Connective differences between patients with depression with and without ASD: A case-control study
Source: PLoS One. 2023 Aug 15;18(8):e0289735. doi: 10.1371/journal.pone.0289735 (PMC10427005; doi:10.1371/journal.pone.0289735)
Supplement: Attachment — Submitted filename: reply_to_author__wo_ja.docx [file pone.0289735.s004.docx]

Dear Dr. Federico Giove,

Thank you for giving us the opportunity to submit a revised draft of our manuscript titled “Connective differences between patients with depression with and without ASD: a case-control study” to *PLOS One*. We appreciate the time and effort that you and the reviewers have dedicated to providing your valuable feedback on our manuscript. We are grateful to the reviewers for their insightful comments on my paper. We have been able to incorporate changes to reflect most of the suggestions provided by the reviewers. We have highlighted the changes within the manuscript.

Here is a point-by-point response to the reviewers’ comments and concerns.

1.Authors pls expalin what is the dfifference between depressive state and depression.? Does the authors trying to find out mild , moderate or severe level of depression?

Response: Thank you for reviewing my manuscript.

Skilled psychiatrists examine patients using the DSM5 diagnostic criteria. Based on these diagnostic criteria, depression is diagnosed as a disease, and not as a depressive state. We have revised the definition of the disease and have modified the description of ASD with depressive state to Depression with ASD.

　The association between depression severity and ASD is not directly relevant to the purpose of this study. Considering the data obtained in the course of our review, we regarded it appropriate to remove the study item that classifies based on the severity of the depressive state.

2.The authors have mentioned the preprocessing methods such as slicing, band pass filtering, smoothing and normalization but there is axial information of the filtered image , sliced image or normalized image ?Probably you can tabulate the results of each step and its S/N ratios achieved.

Response: We calculated the signal-to-noise ratio (SNR) for the images before preprocessing and after realignment, normalization, and smoothing. fMRI is used for analyzing neural activity-induced signal changes in the cerebral cortex. Signal changes are caused by neural activity in the cortex. Thus, the analysis was performed using segmented white matter and the conventional method of SNRs, which utilizes the four corners of the image as noise.

　The results suggest that preprocessing improves the SNR of fMRI.

3.Though you use CONN connectivity tool box , need to mention the actual analysis between depressive state and depression this analysis. Pls give the raw image of Depression and Depressive state image.

Response:　In the previous revision, we had been asked to depict the flow of the image analysis; thus, we added an analysis flow diagram. In this study, we added the flow of the 1^st^ and 2^nd^ level analysis.

In this study, we did not obtain consent from the participants to release their images. Because several participants were terminally end of medical examination, we were unable to obtain an additional consent for release.

4 Authors claim that the statistical analysis by performing the F test, has right supramarginal gyrus (SMG) (p-FDR < 0.04*) and decreased connectivity to the left hippocampus (p-FDR < 0.02*) and para-hippocampus (p-FDR < 0.02*). What tool is used for this test ?.What are the hypothesis? Pls mention the Mean , STD from the statistical analysis.

Response: During this revision stage, we have added the following text regarding the process of analysis at the first and second levels. We used the general linear model as the analysis method for the second level and have added references for the detailed analysis. We have mentioned that we used the multiple comparison correction employed in the CONN to avoid the first type of error when comparing each voxel. We performed significance testing with FDR as a multiple comparison correction at the cluster level to assess significantly higher or lower ROI connectivity in patients with ASD, compared with healthy controls.

　In this study, we did not obtain consent from the participants to release their images. Because several participants were terminally end of medical examination, we were unable to obtain an additional consent for release.

5.Pls check the entire manusript for phraseaology, grammar ,and the connectivity between each section.

6.Improve the discussion section

Response: Because we are not native speakers, we have used the English editing service by Editage and have received certification.

　We have made the necessary changes and have added references where the statements were incoherent.
