# Supplementary material for: Connective differences between patients with depression with and without ASD: A case-control study
Source: PLoS One. 2023 Aug 15;18(8):e0289735. doi: 10.1371/journal.pone.0289735 (PMC10427005; doi:10.1371/journal.pone.0289735)
Supplement: Attachment — Submitted filename: reply_to_author_wo_ja_0717.docx [file pone.0289735.s005.docx]

Dear Dr. Federico Giove,

Thank you for giving us the opportunity to submit a revised draft of our manuscript titled “Connective differences between patients with depression with and without ASD: a case-control study” to PLOS One. We appreciate the time and effort that you and the reviewers have dedicated to providing your valuable feedback on our manuscript. We are grateful to the reviewers for their insightful comments on my paper. We have been able to incorporate changes to reflect most of the suggestions provided by the reviewers. We have highlighted the changes within the manuscript.

Here is a point-by-point response to the reviewers’ comments and concerns.

Journal Requirements:

1. The manuscript is fine, it can be accepted from a scientific standpoint.

The data access policy stated in the manuscript is not acceptable. Authors must report in the article text the same policy declared in the forms, and in particular name and email of the person to be contacted to get access to the data.

Response: Thank you for reviewing my manuscript.

We have changed our data access policy in the manuscript to be fit in the form.

2. Please review your reference list to ensure that it is complete and correct. If you have cited papers that have been retracted, please include the rationale for doing so in the manuscript text, or remove these references and replace them with relevant current references. Any changes to the reference list should be mentioned in the rebuttal letter that accompanies your revised manuscript. If you need to cite a retracted article, indicate the article’s retracted status in the References list and also include a citation and full reference for the retraction notice.

Response: We have added "doi" to the No. 35 reference. In addition, we changed the style of No. 36 to that of the Book.

There were no comment from reviewer. So, we have submitted a revised version of the paper we submitted on July 9.
